# Age and sex affect deep learning prediction of cardiometabolic risk factors from retinal images

**DOI:** 10.1038/s41598-020-65794-4

**Published:** 2020-06-10

**Authors:** Nele Gerrits, Bart Elen, Toon Van Craenendonck, Danai Triantafyllidou, Ioannis N. Petropoulos, Rayaz A. Malik, Patrick De Boever

**Affiliations:** 1 VITO NV, Unit Health, Mol, Belgium; 2 Weill Cornell Medicine - Qatar, Doha, Qatar; 3 Hasselt University, Diepenbeek, Belgium; 4 Department of Biology, University of Antwerp, Universiteitsplein 1, 2610 Wilrijk, Belgium

**Keywords:** Predictive markers, Risk factors

## Abstract

Deep neural networks can extract clinical information, such as diabetic retinopathy status and individual characteristics (e.g. age and sex), from retinal images. Here, we report the first study to train deep learning models with retinal images from 3,000 Qatari citizens participating in the Qatar Biobank study. We investigated whether fundus images can predict cardiometabolic risk factors, such as age, sex, blood pressure, smoking status, glycaemic status, total lipid panel, sex steroid hormones and bioimpedance measurements. Additionally, the role of age and sex as mediating factors when predicting cardiometabolic risk factors from fundus images was studied. Predictions at person-level were made by combining information of an optic disc centred and a macula centred image of both eyes with deep learning models using the MobileNet-V2 architecture. An accurate prediction was obtained for age (mean absolute error (MAE): 2.78 years) and sex (area under the curve: 0.97), while an acceptable performance was achieved for systolic blood pressure (MAE: 8.96 mmHg), diastolic blood pressure (MAE: 6.84 mmHg), Haemoglobin A1c (MAE: 0.61%), relative fat mass (MAE: 5.68 units) and testosterone (MAE: 3.76 nmol/L). We discovered that age and sex were mediating factors when predicting cardiometabolic risk factors from fundus images. We have found that deep learning models indirectly predict sex when trained for testosterone. For blood pressure, Haemoglobin A1c and relative fat mass an influence of age and sex was observed. However, achieved performance cannot be fully explained by the influence of age and sex. In conclusion we confirm that age and sex can be predicted reliably from a fundus image and that unique information is stored in the retina that relates to blood pressure, Haemoglobin A1c and relative fat mass. Future research should focus on stratification when predicting person characteristics from a fundus image.

## Introduction

Mortality from cardiovascular disease (CVD) remains the leading cause of death worldwide today^1,2^ and is an increasing burden of CVD in the Middle East^3–8^, with premature myocardial infarction and stroke. This CVD burden may be reduced by the early identification of individuals on a trajectory to develop the disease by providing a program of lifestyle changes and medication to alter the course of the disease. Therefore, tools for CVD risk stratification are crucial in identifying at-risk individuals. The Framingham score was one of the first tools to estimate individual CVD risk in the US using low-cost variables such as age, sex, smoking status, cholesterol and blood pressure^9^. This allows targeting of healthcare resources to those likely to benefit from preventive medical care and avoid possible adverse outcomes and costs of unnecessary care in those at low risk^10^. Scores such as the Framingham score, SCORE charts^11^, the QRISK models^12^ and U-prevent tool^13^, have reduced prediction accuracy in individuals with different ethnicity than the ones that were used to develop the score^14–17^. Indeed, there are no risk scores developed in populations from the Middle East^18^. Additionally, the limited number of risk indices used as input parameters oversimplify the complex pathogenesis of CVD development^19^ and at best predict 30% of the risk.

In the search for identifying new methodologies to optimize the performance of risk prediction, Weng *et al*. trained machine learning classifiers on data from 30 variables associated with CVD from 378,256 UK citizens to predict a first cardiovascular event over 10 years and demonstrated a marked improvement in cardiovascular risk prediction when compared to established risk factors^19^. Similarly, Ambale-Venkatesh *et al*. trained random forests and showed enhanced prediction of six cardiovascular outcomes, including stroke and heart failure using 735 variables from imaging and noninvasive tests, questionnaires and biomarker panels. Interestingly, imaging, electrocardiography and serum biomarkers featured heavily in the top-20 of the random forests, as opposed to traditional cardiovascular risk factors^20^.

Retinal image analysis quantifying retinal vessel metrics may provide additional information for CVD risk stratification. Typically, investigators report links between hand-crafted features, such as vessel widths, derived from a fundus picture and a variety of cardiometabolic risk factors and diseases^21^. Ding and coworkers have shown that narrower retinal arterioles and wider venules are associated with an increased 10-year risk of hypertension^22^. Furthermore, in a recent study by Owen *et al*. atherosclerotic risk factors were correlated with arteriolar width^23^. Seidelmann *et al*. have shown that narrower retinal arterioles and wider venules are associated with increased mortality and ischemic stroke in both sexes and coronary heart disease in women^24^. Even though retinal imaging has been accepted as a promising imaging modality to assess the development of cardiovascular diseases, its ability to be applied in CVD risk score estimators has yet to be evaluated.

Recently, deep learning, a form of artificial intelligence, has been introduced for extracting more information from retinal images. The landmark paper of Gulshan and coworkers has been transformative because it showed that a deep learning algorithm can detect referable diabetic retinopathy with high sensitivity and specificity^25^. Along the same lines, Poplin *et al*. found that several risk factors, such as age, sex, smoking status and blood pressure, can be predicted directly from fundus images using deep neural networks^26^. More recently, Vaghefi *et al*. trained a convolutional neural network for the prediction of smoking status using fundus images only^27^. These two recent studies suggest that deep learning can rapidly extract more detailed information from fundus images to aid in CVD risk determination. However, both studies have not included relevant clinical measurements for CVD risk estimation such as the total lipid panel^28^.

Our study builds on the work of Poplin *et al*. and Vaghefi *et al*. and contributes in two key ways. We include a wider set of cardiometabolic and clinical measurements to obtain a more complete picture of the confounders and predictors of retinal image parameters extracted using deep learning, especially in relation to age and sex. Furthermore, this is the first study to study a Middle-Eastern population by utilizing retinal images from the Qatar Biobank.

## Results

### Study population

Demographics of the individuals included in the study, which comprise a subset from the Qatar Biobank initiative^29^, are summarized in Table 1. The mean age was 40.6 ± 13.0 years. 20% of all participants (26% of men; 16% of women) had hypertension based on a systolic blood pressure (SBP) higher than 130 mmHg or a diastolic blood pressure (DBP) higher than 80 mmHg, according to the guidelines of the American Heart Association^30^. The incidence of overweight and obese persons in the study population was 72% in men and 71% in women.

**Table 1:** Descriptive statistics of individuals in this subset of the Qatar Biobank data.

Characteristics	Qatar Biobank subset
Number of participants	3,000
Number of images	12,000
Age (years)	40.6 (13.0)
Sex (% male)	41%
Ethnicity	86% Qatari, 14% mix of 25 other countries
Current smoker (%)	18%
Body Mass Index (kg/m2)	29.7 (6.4)
Relative fat mass (%)	38.4 (9.9)
Systolic blood pressure (mmHg)	114.4 (15.6)
Diastolic blood pressure (mmHg)	66.4 (10.0)
Haemoglobin A1c (%)	5.8 (1.4)
Insulin (mcunit/ml)	13.3 (16.6)
Glucose (mmol/L)	6.0 (2.5)
Sex hormone binding globulin (nmol/L)	52.4 (43.4)
Estradiol (pmol/L)	254.6 (314.4)
Testosterone (nmol/L)	7.9 (9.3)
Total cholesterol (mmol/L)	5.0 (1.0)
HDL cholesterol (mmol/L)	1.4 (0.4)
LDL cholesterol (mmol/L)	3.0 (0.9)
Triglyceride (mmol/L)	1.3 (0.9)

For numerical variables the mean and standard deviation are shown.

### Prediction of cardiometabolic risk factors

First, we predicted age and sex from available fundus images using deep learning. Performance results achieved on the test set include a mean absolute error (MAE) of 3.21 years (95% confidence interval (CI): 3.07 to 3.36) and a coefficient of determination (*R*^2^) of 0.85 (95% CI: 0.83 to 0.87) for predicting age; and an area under the curve (AUC) of 0.96 (95% CI: 0.95 to 0.97) with an accuracy of 0.90 (95% CI: 0.89 to 0.91) for predicting sex. A boost in the model’s performance was observed when carrying out the evaluation on person level by taking into account the information from the four fundus images that were available per person. Practically, this was obtained by averaging the output of the model for the four different input images. This was confirmed by a *R*^2^ and MAE on the test set of 0.89 (95% CI: 0.86 to 0.92) and 2.78 years (95% CI: 2.55 to 3.05) on person level for age prediction. The same observation was made for sex prediction, where an evaluation on person level reached an accuracy of 0.93 (95% CI: 0.91 to 0.95) and an AUC of 0.97 (95% CI: 0.96 to 0.98). Since performance increased by evaluating on person level instead of image level, all further experiments are reported on person level.

In a second series of experiments we developed deep learning models to predict a series of cardiometabolic risk factors, namely blood pressure, smoking status, diabetes-related tests, lipid panel, and bioimpedance measurements. For the purpose of investigating the impact of sex on the model’s performance, sex steroid hormones are added to the list as well.

**Figure 1:**
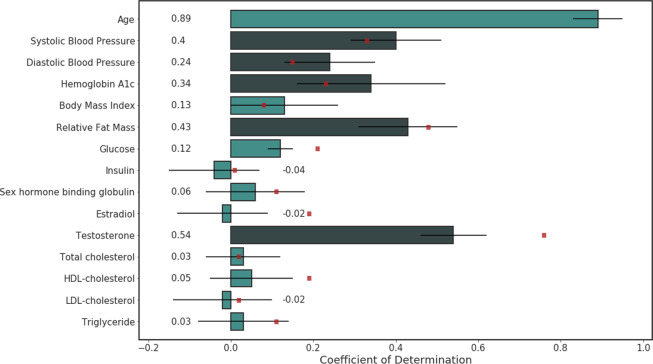
Model performance on predicting continuous cardiometabolic risk factors in the test set on person level for the regression task. The coefficient of determination is plotted for every risk factor, along with the 95% confidence interval obtained via a bootstrapping methodology. Results for a linear regression using age and sex on person level on the test set is added for every cardiometabolic risk factor, except age, with a red dot. Risk factors included in the exploration of the impact of age and sex on prediction performance have a coefficient of determination higher than 0.20 and are indicated in dark green.

Figure 1 shows an overview of the obtained performance metrics for predicting the cardiometabolic risk factors on person level for the regression task. Several metrics reached relatively good performance, including SBP (${R}^{2}=0.40$, $MAE=8.96$ mmHg), DBP (${R}^{2}=0.24$, $MAE=6.84$ mmHg), Haemoglobin A1c (HbA1c) (${R}^{2}=0.34$, $MAE=0.61 \%$), relative fat mass (${R}^{2}=0.43$, $MAE=5.68$ units) and testosterone (${R}^{2}=0.54$, $MAE=3.76$ nmol/L), whilst others obtained poor performance, including the total lipid panel (${R}^{2}\le 0.05$). In addition, a prediction model for smoking status achieved an accuracy of 0.81 and an AUC of 0.78 on person level. Detailed results, including 95% confidence intervals, are listed in Table 1 in the Supplementary Information.

### Impact of age and sex on prediction performance

Our initial results showed that age and sex could be predicted from fundus images with high precision. For this reason, the possibility that the trained deep learning architecture was reporting inherent correlations among age, sex and the target variable was examined. This is specifically done for variables that could be predicted with a *R*^2^ higher than 0.20 (regression task) and an AUC higher than 0.80 (classification task).

First, the predictive value of variables age and sex to predict the clinical variable was investigated by constructing simple linear regression models with age and sex as independent variables (Table 1 in the Supplementary Information, Fig. 1). Relatively good performance metrics were achieved with a linear regression model for clinical variables such as blood pressure, HbA1c, relative fat mass and testosterone. For blood pressure, HbA1c, BMI and total cholesterol the deep learning model based on fundus images reached a higher *R*^2^ than the linear regression. All other variables obtained a lower *R*^2^ than the linear model.

**Figure 2:**
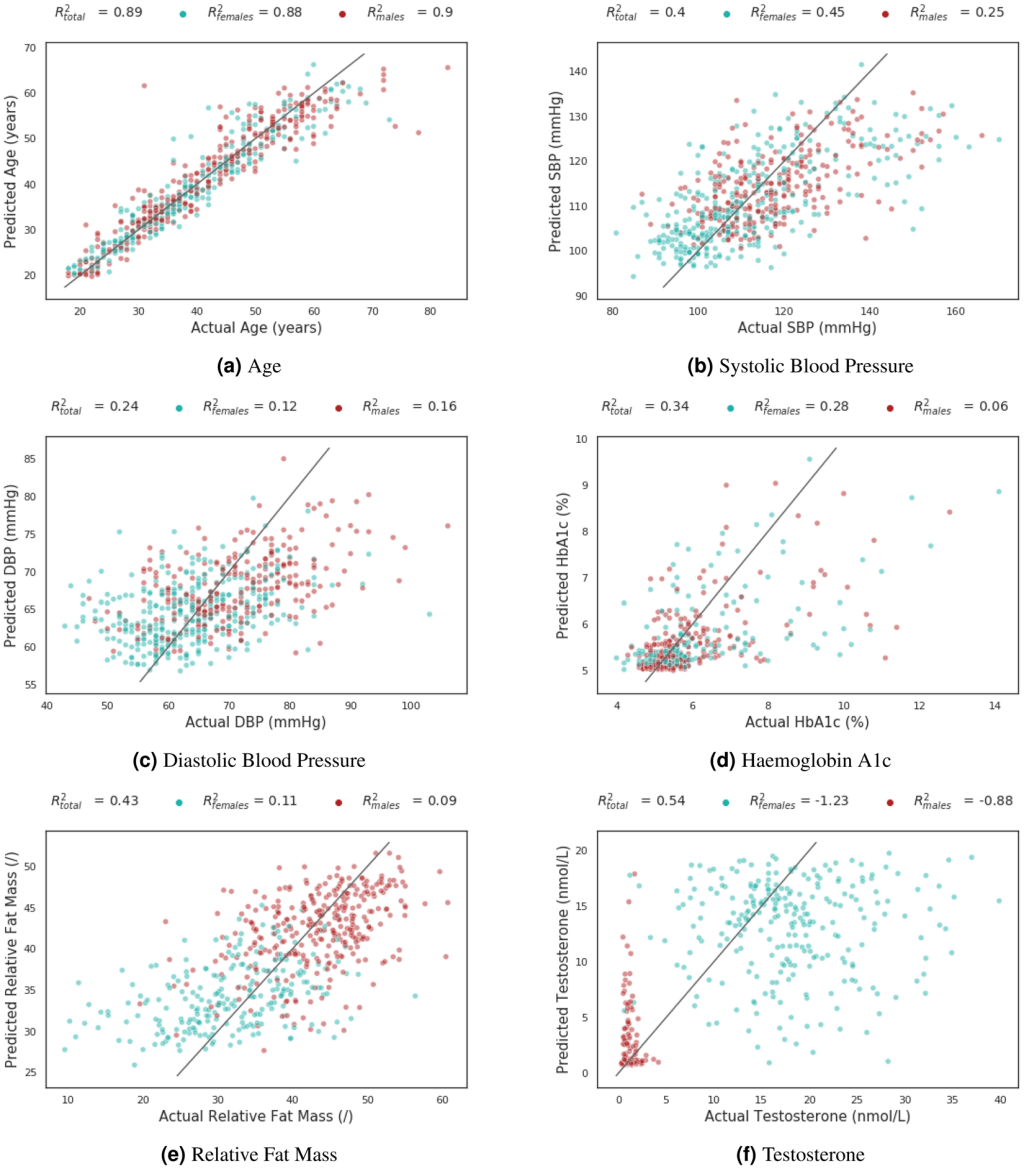
Predicted and actual value for the test set for an evaluation on person level for several cardiometabolic risk factors, stratified as per sex (female is coloured blue and male is coloured red). Units for age are years (left upper), for SBP are mmHg (right upper), for DBP are mmHg (middle left), for HbA1c are % (middle right), for relative fat mass are units (lower left) and for testosterone are nmol/L (lower right). The lines represent y = x values.

To investigate this further, the test set was split according to sex and age, such that an approximately equal number of participants were found in each age group (155 persons < 30 years, 163 persons ≥ 30 years and <39 years, 130 persons ≥ 39 years and <50 years, 152 persons ≥ 50 years). Differences in these subpopulations were examined for the impact of age and sex. First, the model’s performance on the test set was stratified by age and sex and the differences among groups were compared. The training and validation set, as well as the trained model, were kept unchanged for this experiment. Results for this analysis are presented in Fig. 2 for sex and in Table 2 for age. For sex, all results are also listed in Table 2 in the Supplementary Information.

**Table 2:** Performance of the algorithm stratified per age category on person level.

	<=30 years	>30 and <=39 years	>39 and <=50 years	>50 years	Total
Sex (% male)	0.99	0.96	0.98	0.97	0.97
Systolic blood pressure (mmHg)	0.24	0.23	0.30	0.04	0.40
Diastolic blood pressure (mmHg)	0.16	0.16	0.21	0.21	0.24
Haemoglobin A1c (%)	−0.14	0.28	0.24	0.06	0.34
Relative fat mass value	0.29	0.33	0.33	0.47	0.43
Testosterone (nmol/L)	0.61	0.57	0.45	0.50	0.54

Performance is given by showing *R*^2^ (regression task) or the AUC (classification task) on the test set.

As can be seen (Fig. 2 and Table 2 in the Supplementary Information) considerable differences in model performance were found between females and males for testosterone and relative fat mass. This may imply that the same visual characteristics on the fundus image that are important for sex prediction are now also employed by the model, indicating that the model may be indirectly predicting sex. Furthermore, there were no differences in model performance between females and males for age and HbA1c, indicating that visual characteristics other than those important for explaining sex are now expected to be employed by the deep learning model. In Subfigure 2a the data points for the prediction of age of females and males are equally distributed around the diagonal and no clear trend can be seen. For testosterone, however, it can be seen in Subfigure 2f that the majority of the female data points lie in the left lower corner, which can be explained by the lower female testosterone values, and that the majority of the male data points are situated in the upper right corner. To a lesser extent than for testosterone two clusters are noticed on Subfigures 2b, 2c and 2e representing the predictions for females and males, indicating some influence of sex on performance. For HbA1c no clear tendency is noticed in the model performance for the female and male groups in Subfigure 2d.

A similar analysis can be made on the results displayed in Table 2, where differences in model performance between the age groups stand out for HbA1c and SBP. No differences in model performance were found for sex and diastolic blood pressure. Age seemed to influence the model’s performance for testosterone and relative fat mass.

Overall, these results suggest that the models predicting certain cardiovascular risk factors other than age and sex pay attention to the same visual characteristics already explaining age and/or sex. Although the results of the latter experiment allow us to observe trends, it is difficult to draw strong conclusions. To illustrate (Subfigure 2f), testosterone is linked to sex, but the directionality of this relationship is not straightforward. The deep learning model for sex relies on visual characteristics on the fundus explaining the testosterone value, or the opposite could be true. Since the performance for the sex prediction was markedly higher than the prediction for testosterone based on the fundus images, it suggests that the model inherently predicts sex when trained for testosterone.

To further examine the impact of sex on the prediction of cardiometabolic risk factors, a final experiment was conducted. Deep learning models were trained on fundus images of females (7,080 images in total) and males only (4,920 images in total) for the prediction of the six clinical variables in the previous experiment. This was done for sex since it is a binary variable and groups are easily made, whilst for age this is not feasible. As sample size decreases considerably, it is not fair to compare the obtained performances with the one on the total dataset. To have a fair baseline, a deep learning model was trained on a random sample containing half of the total dataset (Table 3). For testosterone, *R*^2^ decreased from 0.54 to 0.48 on person level due to the smaller sample size. For age, *R*^2^ slightly decreased from 0.89 to 0.86 on person level. When training on the female subset, an *R*^2^ of 0.03 and 0.89 was achieved for testosterone and age, respectively. When training on the male subset, an *R*^2^ of 0.04 and 0.86 was achieved on the test set for testosterone and age, respectively. This result supports the hypothesis that the deep learning model indirectly predicts sex when it is trained for testosterone prediction, as performance breaks down when trained only for females and males. It also shows that the deep learning model for age prediction does not rely on the sex prediction since performance remained almost constant over all different groups. For SBP, DBP, HbA1c and relative fat mass an influence of sex was observed.

**Table 3:** Performance results for training on subsets of the data, on females, on males and on a random half subset of the Qatar Biobank data.

	Females	Males	On 1/2 training set	On total training set
Age (years)	0.89	0.86	0.86	0.89
Systolic blood pressure (mmHg)	0.44	0.19	0.29	0.40
Diastolic blood pressure (mmHg)	0.14	0.21	0.14	0.24
Haemoglobin A1c (%)	0.28	0.16	0.25	0.34
Relative fat mass	0.23	0.07	0.40	0.43
Testosterone (nmol/L)	0.03	0.04	0.48	0.54

For completeness the performance results on the test set when trained on the total training set are included in the table as well. Obtained *R*^2^ values on the respective test sets on person level are shown.

## Discussion

This paper reports on deep learning applied to fundus images in order to predict cardiometabolic risk factors such as age, sex and blood pressure, HbA1c, lipid panel, sex steroid hormones and bioimpedance measurements. We show that high performance results are achieved for age and sex, both important cardiovascular risk factors, and to a lesser extent for other cardiometabolic risk factors, such as blood pressure, HbA1c and relative fat mass. Our experiments suggest that age and sex are mediating factors in the prediction of the latter risk factors, meaning that the deep learning models to some extent indirectly predict age and/or sex. However, unique information can be found in the retinal image that relates to blood pressure, HbA1c and relative fat mass.

We obtained an *R*^2^ of 0.89 for predicting age on person level vs. 0.74 on the UK BioBank and 0.82 on the EyePACS data for image level by Poplin *et al*. and an AUC of 0.97 for predicting sex on person level vs. 0.97 on image level by Poplin *et al*. Performance increase was observed in our study when multiple fundus images of both eyes and multiple centre modes were employed for a single prediction, indicating that the use of more sophisticated methods of combining different fundus images could improve performance significantly. Additionally, when comparing performance results for other clinical biomarkers to Poplin *et al*. SBP and BMI achieved a similar performance, whilst DBP and smoking status achieved a somewhat lower performance.

For HbA1c we report a significantly higher performance than Poplin *et al*. with an *R*^2^ of 0.34 on person level vs. 0.09 on image level on the EyePACS data^26^. A possible explanation is a difference in the population examined as we used a Middle-Eastern population, whereas Poplin *et al*. examined a population of diabetic patients who were primarily white and middle-class with a higher mean HbA1c value and a wider distribution for their study population (8.2% ± 2.1 vs. 5.8% ± 1.4). Another difference is the higher mean age in Poplin’s population ($53.6\,yrs\pm 11.6$ vs. $40.6\,yrs\pm 13.0$) while we have shown that the performance of the HbA1c prediction is influenced considerably by a person’s age (see Table 2).

For the first time deep learning models were trained for the prediction of the lipid panel (total cholesterol, HDL- and LDLcholesterol, and triglyceride) and glycaemic markers (glucose and insulin) on fundus images. Performance was found to be poor, however the samples from the Qatar Biobank were non-fasting glucose and the participants were taking various glucose and lipid lowering medications.

For smoking status Vaghefi *et al*. achieved (AUC 0.86) a significantly higher performance compared to Poplin *et al*. (AUC 0.71 on UK Biobank) and our data (AUC 0.78). A possible explanation is the considerably higher number of smokers in our dataset (18% versus 10% in Poplin *et al*. and 9% in Vaghefi *et al*.). Also, smoking habits in the Qatar Biobank population were established from four questions about a person’s smoking habit as opposed to a single question in other studies.

Despite the notorious data-hunger of deep learning, we obtained comparable predictive performances for most risk factors in a smaller dataset (3,000 participants) compared to the EyePACS and UK Biobank datasets (284,335 participants)^26^ and the Australian diabetic screening program (81,711 participants)^27^. Reasons for this include the application of transfer learning and data augmentation and by aggregating information from multiple retinal images per person. Furthermore, fundus images were taken with one type of fundus camera at the same physical location, namely the Qatar Biobank facility, in a relatively homogeneous population of Qatari citizens. Additionally, the clinical investigations were undertaken by a limited number of trained investigators, leading to a low operator bias. The database of the Qatar Biobank is considered more standardized and homogeneous than the UK Biobank or EyePACS datasets. These have been collected from multiple measurement centres or with different types of fundus cameras, and with a larger pool of operators. Therefore, it is anticipated that the generalization to other populations is more limited than in the case of Poplin *et al*.^28^. Yet, generalisation is a necessary key challenge in deep learning^31,32^. It is important to note that the goal of the current study was to investigate the proof-of-principle of deep learning for the prediction of cardiometabolic factors from fundus images and to identify confounders when predicting these factors. However, future work is needed on generalisation of our findings.

Since age and sex can be predicted with high precision from fundus images, we analysed whether the model is indirectly predicting sex and/or age instead of the target variable. For the clinical variables showing no considerable differences in performance when stratifying for age and sex, the models are expected to be not indirectly predicting age and/or sex and instead looking for unique signs in the retina explaining the target variable. For the variables with divergent *R*^2^ for the different categories (sex and age groups) it is hypothesized that the model may be indirectly predicting age and/or sex and thus looking for similar signs in the retina. This raises the question as to whether the fundus image captures additional information regarding the clinical biomarkers, or whether it is simply basing its prediction on age and sex. This allowed us to assess hidden stratification, similar to Oakden-Rayner *et al*.^33^, especially as we observed a lower performance in other biomarker subclasses when stratifying for age and sex. Models were trained on females and males only to assess whether sex is a mediating factor in the prediction of certain clinical variables. The model predicting testosterone was inherently predicting sex, but the model predicting age was not based on the prediction of sex. For other cardiometabolic risk factors this relationship was not as clear as for age and testosterone.

This paper is the first to apply deep learning for predicting cardiometabolic risk factors in a Middle-Eastern population that typically remain understudied. Detailed phenotyping was undertaken for each study participant in a consistent manner following standard protocols. Our dataset includes the lipid panel which is highly relevant for CVD risk prediction, noted as a major limitation of the study by Poplin *et al*.^28^. In addition, we utilized a light-weight architecture requiring much less computer power when compared with the Inception-v3 model used by Poplin *et al*.^34^. To the best of our knowledge, this study is the first comprehensive investigation assessing the effect of age and sex on the performance of prediction models for cardiometabolic risk factors based on fundus images. The data is from a Middle-Eastern population, which limits the generalizability of our findings.

## Conclusion

To summarize, this is the first proof-of-concept study from the Middle East which demonstrates that deep learning models predicting cardiometabolic risk factors on fundus images reveal outstanding performance for age and sex, and an acceptable performance for blood pressure, HbA1c, relative fat mass and testosterone. Since age and sex appear to be strong mediating factors when predicting certain cardiometabolic risk factors from retinal images, further studies should focus on understanding the mechanism behind age and sex prediction on fundus images.

## Methods

### Study population

The study population is a subset taken from the Qatar Biobank initiative, a cohort aiming to prospectively examine 60,000 Qataris and long term Qatar residents. Study participants underwent extensive questionnaires (concerning lifestyle, health behaviours, etc.), as well as anthropometric and clinical evaluation (blood pressure, electrocardiogram, etc.). The current study had access to clinical features and questionnaires taken on 3,000 Qatari citizens, summarizing physical, clinical and biochemical measurements. As smoking shisha (waterpipe) is as harmful as smoking cigarettes^35^, it was added to the definition of smoker. Since WHO/ISH have defined smokers as current smokers and people who have stopped smoking less than one year ago^36^, the age when stopped smoking is also taken into account. Details on the smoking definition can be seen in Fig. 1 in the Supplementary Information.

For a detailed description concerning the clinical measurements and protocols used the reader is referred to Kuwari *et al*.^29^. The Qatar Biobank study is conducted according to the regulations and guidelines for Research Involving Humans of the Qatar Ministry of Public Health. Written informed consent was obtained from all participants and approval was received from the Ethics Committee of the Institutional Review Board Hamad Medical Corporation in Doha, Qatar.

### Fundus photography

45° macula- and optic disc centred retinal photographs of the left and right eye were taken using a Topcon TRC-NW6S non-mydriatic retinal camera^29^. The fundus image resolution was 1600 × 1059.

### Model development

Since four fundus images were available per person, a total of 12,000 fundus images were accessible and were randomly divided into a training (60%), validation (20%) and test set (20%), ensuring that fundus images of the same person were present in the same split. In total, 7,200 images were used for training and the models were validated and tested on two sets each containing 2,400 images. Missing values were observed for BMI (n = 265), RFM (n = 265), HbA1c (n = 2), insulin (n = 9), Sexhormone binding globulin (n = 134), estradiol (n = 266), testosterone (n = 15), HDL-cholesterol (n = 3) and LDL-cholesterol (n = 33). Whenever an image had a missing value for the variable we were training for, it was not used in the training process. For the prediction of smoking status oversampling of the minority class has been used during the training process to mitigate the class imbalance of 529 smokers, versus 2,471 non-smokers. Oversampling was applied only on the training set where smokers were sampled three times resulting in a balanced training set that contained 1,244 smokers and 1,489 non-smokers. The observed imbalance remained unchanged in the validation and test set.

After empirically testing different image sizes and preprocessing functions, a pipeline was selected based on the achieved performance. All images were rescaled to 400 × 400 pixels and were pre-processed following a pipeline that was used successfully before for deep learning applied on retinal images^37^. With a Gaussian filter the local average colour was subtracted and mapped to 50% grey and the images were clipped to 90% of their size to remove the boundary effect. Several data augmentation techniques were also applied. In particular, the training images were horizontally flipped, randomly rotated from 0 to 360 degrees and randomly shifted by 10% in height and width. No data augmentation was applied to the test set. Batch size was 32.

We empirically found that the MobileNet-V2 architecture was well fitted for our study. We tried other popular network architectures for which pretrained weights are available in the Keras library such as ResNet and VGG16, but did not get better results and they were slower to train^38,39^. The MobileNet architectures are based on depth wise separable convolutions to maintain a light weight and efficient deep learning model^39,40^. A MobileNet-V2 architecture pretrained for the ImageNet competition without top layers with input size 400 × 400 × 3 represents the baseline model in this paper. The prediction of continuous variables such as age was handled as a regression task for which a global average pooling layer and two fully connected layers were added to the baseline. The first fully connected layer had 512 neurons and a Rectified Linear Unit (ReLU) activation and the last layer had one output and a linear activation. For the prediction of the categorical variables such as sex a second model was trained, which adds a global average pooling layer and two fully connected layers to the baseline. The first fully connected layer had 512 neurons and a ReLU activation and the last layer had one output and a sigmoid activation. No layers were frozen during the training process and the training process was identical for all different cardiometabolic risk factors. Information from the four available fundus images per person was aggregated by averaging the output of the deep learning model for these four images.

Regression models were optimized by minimizing the MAE between the predicted and the actual values of the predicted variable. Classification models were optimized by minimizing the binary cross-entropy loss. The models were trained for 150 epochs using the Adam optimization algorithm using the default parameters provided in the original paper^41^. Early stopping was applied by interrupting training of the model when validation loss did not improve in the last 50 epochs. The model with the best validation performance during all runs was saved and used. TensorFlow 1.4.0 and Keras 2.2.4 open-source software libraries were used for the creation of the deep learning models.

To evaluate model performance for continuous variables (regression task) the MAE and *R*^2^ were computed. For classification tasks such as sex prediction the accuracy and AUC were computed.

### Statistical analysis

To assess robustness of performance metrics, a bootstrap approach is applied. First, performance is computed on 2000 random samples with replacements obtained from the test set containing as much samples as the test set. Secondly, a mean value and 95% confidence interval is computed on the resulting distribution.

In order to have a baseline for the predictive power of age and sex for the clinical variables, multiple linear regression models with age and sex as independent variables were trained to predict these variables. All statistical analyses were performed in python using the Statsmodels 0.10.1, Pandas 0.21.0 and Scikit learn 0.19.1.

## Supplementary information

Supplementary Information.
