# Publisher Correction: Age and sex affect deep learning prediction of cardiometabolic risk factors from retinal images

**DOI:** 10.1038/s41598-020-80629-y

**Published:** 2021-01-07

**Authors:** Nele Gerrits, Bart Elen, Toon Van Craenendonck, Danai Triantafyllidou, Ioannis N. Petropoulos, Rayaz A. Malik, Patrick De Boever

**Affiliations:** 1 VITO NV, Unit Health, Mol, Belgium; 2 Weill Cornell Medicine - Qatar, Doha, Qatar; 3 Hasselt University, Diepenbeek, Belgium; 4 Department of Biology, University of Antwerp, Universiteitsplein 1, 2610 Wilrijk, Belgium

Correction to: *Scientific Reports* 10.1038/s41598-020-65794-4, published online 10 June 2020

In the original version of this Article, the author Patrick De Boever was incorrectly indexed. This error has now been corrected.

