# Supplementary material for: Age and sex affect deep learning prediction of cardiometabolic risk factors from retinal images
Source: Sci Rep. 2020 Jun 10;10:9432. doi: 10.1038/s41598-020-65794-4 (PMC7287116; doi:10.1038/s41598-020-65794-4)
Supplement: Supplementary file 1 — Supplementary Information. [file 41598_2020_65794_MOESM1_ESM.pdf]

Age and sex affect deep learning prediction of cardiometabolic risk factors from retinal images Supplementary Information

Nele Gerrits, Bart Elen, Toon Van Craenendonck,
Danai Triantafyllidou, Ioannis N. Petropoulos,
Rayaz A. Malik, Patrick De Boever

	Our results on QBB test set		Linear regression
	MAE/accuracy	R2/AUC	R2/AUC
Age (years)	2.78 (2.55, 3.05)	0.89 (0.86, 0.92)	n/a
Sex	0.93 (0.91, 0.95)	0.97 (0.96, 0.98)	n/a
Systolic blood pressure (mmHg)	8.96 (8.32, 9.58)	0.40 (0.35, 0.46)	0.33
Diastolic blood pressure (mmHg)	6.84 (6.40, 7.29)	0.24 (0.18, 0.30)	0.15
Haemoglobin A1c (%)	0.61 (0.55, 0.69)	0.34 (0.25, 0.42)	0.23
Body mass index (kg/m2)	4.31 (4.02, 4.63)	0.13 (0.06, 0.19)	0.08
Relative fat mass value	5.68 (5.32, 6.04)	0.43 (0.37, 0.48)	0.48
Glucose (mmol/L)	1.06 (0.91, 1.22)	0.12 (0.06, 0.19)	0.21
Insulin (mcunit/ml)	7.15 (5.74, 8.85)	-0.04 (-0.06, -0.03)	0.01
Sex hormone binding globulin (nmol/L)	21.09 (18.90, 23.45)	0.06 (0.01, 0.12)	0.11
Estradiol (pmol/L)	154.18(132.21, 179.03)	-0.03 (-0.09, 0.03))	0.19
Testosterone (nmol/L)	3.76 (3.36, 4.18)	0.54 (0.48, 0.60)	0.76
Total cholesterol (mmol/L)	0.75 (0.71, 0.80)	0.03 (-0.01, 0.07)	0.02
HDL-cholesterol (mmol/L)	0.31 (0.29, 0.33)	0.05 (0.00, 0.09)	0.19
LDL-cholesterol (mmol/L)	0.72 (0.67, 0.77)	-0.03 (-0.08, 0.03)	0.02
Triglyceride (mmol/L)	0.49 (0.44, 0.55)	0.03 (-0.03, 0.09)	0.11
Smoking status	0.81 (0.78, 0.84)	0.78 (0.74, 0.82)	0.79

Table 1: Model performance on predicting cardiometabolic risk factors in the test set on person level (regression and classification task). Results for a linear regression using age and sex on person level on the test set is as well added. For numerical variables the MAE and R^2 are shown. For categorical variables the accuracy and AUC are shown. 95% confidence intervals are shown, which are computed with 2000 bootstrap samples.

	Females		Males		Total
	MAE	R^2	MAE	R^2	R^2
Age (years)	2.86	0.88	2.69	0.90	0.89
Systolic blood pressure (mmHg)	8.99	0.45	8.91	0.25	0.40
Diastolic blood pressure (mmHg)	6.53	0.12	7.24	0.16	0.24
Haemoglobin A1c (%)	0.55	0.35	0.69	0.33	0.34
Relative fat mass value	5.23	0.11	6.28	0.09	0.43
Testosterone (nmol/L)	1.00	-1.23	7.41	-0.88	0.54

Table 2: Performance of the algorithm stratified by sex on person level. For numerical variables the MAE and R^2 are shown.

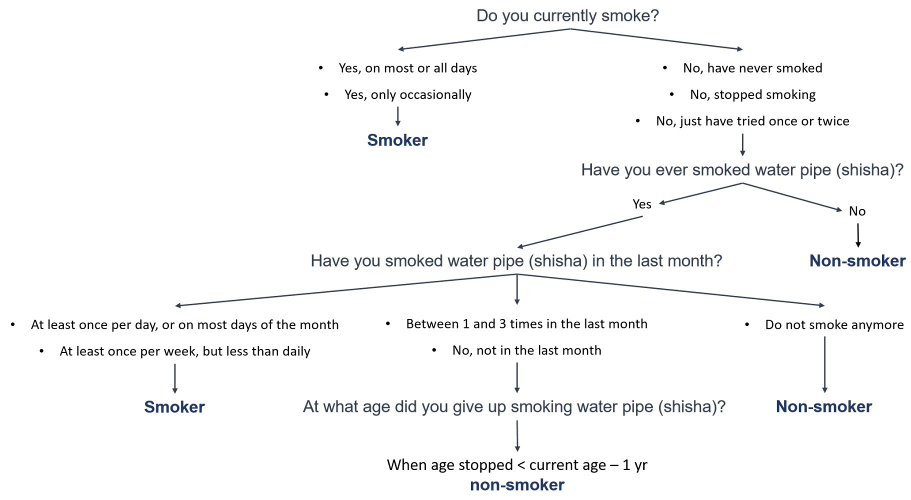

Figure 1: The definition of smoker for the current study based on the main questionnaire of the Qatar Biobank.
